# Quarantine Methods and Prevention of Secondary Outbreak of Pandemic (H1N1) 2009

**DOI:** 10.3201/eid1608.091787

**Published:** 2010-08

**Authors:** Chen-Yi Chu, Cheng-Yi Li, Hui Zhang, Yong Wang, Dong-Hui Huo, Liang Wen, Zhi-Tao Yin, Feng Li, Hong-Bin Song

**Affiliations:** People’s Liberation Army Institute of Disease Control and Prevention, Beijing, People’s Republic of China; 1 These authors contributed equally to this article.

**Keywords:** Outbreak, pandemic (H1N1) 2009, influenza, viruses, quarantine, isolation, China, dispatch

## Abstract

During the 2009 influenza (H1N1) pandemic, some countries used quarantine for containment or mitigation. Of 152 quarantined university students we studied, risk for illness was higher for students quarantined in a room with a person with a confirmed case; we found no difference between students quarantined in double or single rooms.

## Abstract 2

Quarantine Methods and Secondary Outbreak Prevention

Pandemic (H1N1) 2009 influenza emerged in Mexico in March 2009 and by June 10 had rapidly spread to 74 countries (*1**,**2*). Nonpharmaceutical interventions for pandemic influenza at the community level were recommended by the World Health Organization before and during the pandemic (*3**,**4*). One such nonpharmaceutical intervention was quarantine of contacts of persons with confirmed cases. A key question in closed settings (e.g., military barracks) was how to prevent a secondary outbreak of influenza among those quarantined.

The first identified case of pandemic (H1N1) 2009 in mainland People’s Republic of China was imported from the United States and reported on May 11, 2009 (*5*). On May 29, the Chinese Ministry of Health required that each confirmed case-patient and each contact be isolated and quarantined in 1 separate room to contain transmission of the virus (*6*). Containment efforts appeared to successfully prevent community spread until mid-August (*7*). The Chinese Ministry of Health adjusted the quarantine guidelines on August 20 and permitted incomplete quarantine of contacts (e.g., quarantining >1 contact in 1 room) (*8*). We conducted an observational study of a pandemic (H1N1) 2009 outbreak among students of a university in northern China in September 2009. Our goal was to compare the effectiveness of different quarantine methods for preventing a secondary outbreak among the persons in quarantine.

## The Study

During August 31–September 12, 2009, an outbreak of pandemic (H1N1) 2009 occurred among students of a university in northern China. On August 31, pandemic (H1N1) 2009 was laboratory confirmed in 6 students who had fever and acute upper respiratory symptoms. A subsequent investigation found that all of the confirmed case-patients, along with 27 other students, had traveled by train from Shanghai to the university on the afternoon of August 28. One of the students (the index case-patient) had a cough during the trip on August 27. Another 5 students also had fever and influenza-like symptoms and visited the school medical services for treatment during August 28–30. When the outbreak was identified, a total of 202 contacts (average age 21 years, range 19–23 years) were traced and immediately quarantined in a separate dormitory on September 1. Eighty-nine rooms (each with 1 toilet) and 9 apartments (each with 2 bedrooms and 1 toilet) were occupied. One or 2 contacts were assigned to each bedroom. Other control measures, such as ventilating and disinfecting each room, wearing masks, and washing hands, were strictly implemented in accordance with guidance provided by the Chinese Ministry of Health (*8*).

Oropharyngeal swabs from all contacts were collected and tested on the first day of quarantine (September 1). Virologic laboratory testing subsequently indicated that 39 contacts were positive for pandemic (H1N1) 2009. Among the 163 virus-negative contacts, 11 had fever (>38°C) or influenza-like symptoms at the beginning of the quarantine; the other 152 had no fever or influenza-like symptoms on September 1.

Among the 152 virus-negative contacts who had fever or influenza-like symptoms, 20 were exposed to a virus-positive contact during quarantine; 19 shared a bedroom and a toilet with a virus-positive contact; and 1 shared a toilet but not a bedroom with a virus-positive contact. The other 132 were not exposed to any virus-positive contacts during quarantine, including 6 persons housed in single rooms and 126 sharing double rooms.

The temperature and symptoms of contacts were recorded 3× per day (8:00 am, 2:00 pm, and 6:00 pm). We defined a suspected case as a virus-negative contact with sudden onset of fever >38°C and at least 1 of the following symptoms: cough, sore throat, runny nose, shortness of breath, headache, body aches, fatigue, vomiting or diarrhea, and absence of other diagnoses. Students with suspected pandemic (H1N1) 2009 infection who had high fever (>38.5°C) or severe respiratory symptoms (bad cough or dyspnea) were transferred to hospital for treatment; others who had fever <38.5°C or mild influenza-like symptoms were treated in the quarantine building. Because of a shortage of resources, laboratory testing of suspected case-patients in quarantine was not performed after the first day.

Data were analyzed by using SPSS 15.0 (SPSS Inc., Chicago, IL, USA). χ^2^ test and Fisher exact test were used to compare the attack rates of suspected cases between the groups under different quarantine arrangements. A p value <0.05 was considered significant.

During the quarantine, 14 suspected cases were identified among 152 virus-negative contacts, but none had high fever (>38.5°C) or severe respiratory symptoms. Of 19 initially virus-negative contacts sharing a room with a virus-positive contact, 5 suspected cases were identified; onset of symptoms occurred during September 2–8. Also, of 126 initially virus-negative contacts who shared a room with a virus-negative contact, 9 suspected cases were identified; onset of symptoms occurred during September 2–7 (Table, Figure). The attack rate of suspected cases differed significantly between the 2 groups (p = 0.02, 2-tailed Fisher exact test).

**Table:** Evaluation of suspected cases of pandemic (H1N1) 2009 among 152 quarantined persons who were virus-negative at the start of quarantine during an outbreak in northern People’s Republic of China, 2009

Group	Total no.	No. suspected	No. uninfected	p value*
Exposed to virus-positive contacts				
Sharing same room and toilet	19	5	14	0.02†
Sharing same toilet and different room	1	0	1	
Not exposed to virus-positive contacts				
Quarantined 1 to a room	6	0	6	1.00‡
Quarantined 2 to a room	126	9	117	
Total	152	14	138	

*2-tailed Fisher exact test. †p value for the comparison between attack rates of suspected cases among the virus-negative contacts comparing those sharing the same room and toilet with a virus-positive contact and those sharing the same room and toilet with a virus-negative contact. ‡p value for the comparison between attack rates of suspected cases among the virus-negative contacts not exposed to the virus-positive contacts compared with those quarantined in single rooms and those in double rooms.

**Figure:**
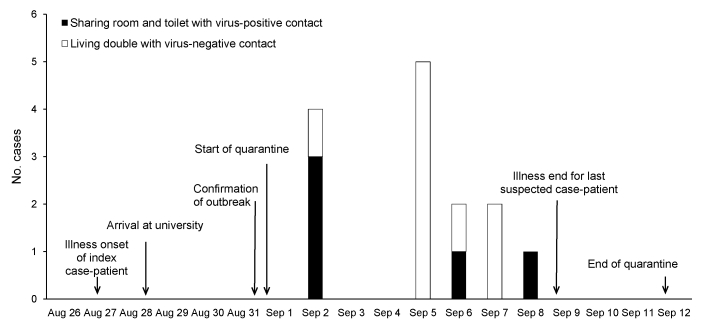
Number of new suspected cases of pandemic (H1N1) 2009 infection per day among 152 quarantined persons who were virus-negative at the start of quarantine during an outbreak in northern People’s Republic of China, 2009.

Among 132 initially virus-negative contacts, who were not exposed to a virus-positive contact during the quarantine, no suspected cases were identified among the 6 students in single rooms, but 9 students with suspected infection were identified among 126 students in double rooms; onset of symptoms occurred during September 2–7 (Table; Figure). Attack rates for suspected cases between the virus-negative contacts in single rooms and those in double rooms did not differ significantly (p = 1.00, 2-tailed Fisher exact test). All students with suspected infection had normal temperature with no influenza-like symptoms after September 9, and all quarantined persons were released by September 12.

## Conclusions

In our study, the attack rate of suspected cases among pandemic (H1N1) 2009 virus–negative contacts increased significantly when persons were quarantined in the same room or used the same bathroom as a virus-positive contact (p = 0.02, 2-tailed Fisher exact test). Nevertheless, quarantining each virus-negative contact in 1 room did not significantly decrease the attack rate of suspected cases among virus-negative contacts in comparison with quarantining 2 persons in 1 room. Our results support the effectiveness of quarantine in preventing a secondary outbreak of pandemic (H1N1) 2009 among contacts of confirmed cases. Our results also support quarantining 2 virus-negative contacts in 1 room in situations where a large number of contacts have been traced but space is limited. Whether our findings support quarantining >2 contacts in 1 room deserves further study.

During the quarantine, every room was routinely disinfected by university staff 1 × per day. Compliance of all contacts with regulations governing personal protection and hygiene was good. Also, staff were assigned to supervise the behavior of contacts in quarantine. These control measures did not contribute to the differences of the attack rate of suspected cases between the different cohorts in this study.

Our study is limited in that virologic laboratory confirmation of suspected cases was not available. However, no other respiratory infections were known to be circulating at that time in the student population, and pandemic (H1N1) 2009 is probably the most likely explanation for influenza-like illness in recent contacts of laboratory-confirmed cases. Lack of laboratory testing also means that we may have underestimated the attack rate during quarantine; some secondary infections may have been associated with asymptomatic or subclinical disease.
